# Prevalence of Hemolysis, Elevated Liver Enzymes, Low Platelet Count Syndrome in Pregnant Women in a Tertiary Care Hospital

**DOI:** 10.31729/jnma.4921

**Published:** 2020-06

**Authors:** Sarita Sitaula, Tara Manandhar, Baburam Dixit Thapa, Ramesh Shrestha, Dinesh Dharel

**Affiliations:** 1 Department of Obstetrics and Gynecology, BP Koirala Institute of Health Sciences, Dharan, Nepal; 2 Department of Pediatrics and Department of Community Health and Epidemiology, University of Saskatchewan, Canada

**Keywords:** *cesarean section*, *HELLP syndrome*, *maternal mortality*

## Abstract

**Introduction:**

Hemolysis, Elevated Liver Enzymes, Low Platelet count syndrome refers to biological syndrome occurring in pre-eclamptic and eclamptic women. There is a higher rate of maternal and perinatal morbidity and mortality due to the syndrome. So, the objective of the study is to find the prevalence and maternal- perinatal outcome in the syndrome.

**Methods:**

A descriptive cross-sectional study was done in a tertiary care hospital from 1^st^ April 2017 to 30^th^ March 2018 after obtaining ethical clearance from the Institutional Review Committee. The inclusion criteria were patients giving consent for participation and those who delivered in our hospital. Patient with the syndrome who delivered outside and referred in the postpartum period was excluded because details of the neonate may not be available. The Statistical Package for Social Sciences version 21 was used for the analysis of the data. Point estimate at 95% Confidence Interval was calculated along with frequency and proportion for binary data.

**Results:**

Out of 11974 deliveries, the prevalence of Hemolysis, Elevated Liver Enzymes, Low Platelet count syndrome was 83 (0.69%) at 95% Confidence Interval (59.06-78.94). Maternal complications were seen in 19 (22.9%) and common complications being acute renal failure 9 (47.37%) followed by postpartum hemorrhage 4 (21.05%). Nearly 27 (33%) of patients required maternal ICU stay and there was one maternal mortality.

**Conclusions:**

Hemolysis, Elevated Liver Enzymes, Low Platelet count syndrome is one of the major causes of maternal and perinatal morbidity and mortality. Hence early recognition and prompt management may improve maternal and fetal outcomes.

## INTRODUCTION

Hemolysis, Elevated Liver Enzymes, Low Platelet count (HELLP) syndrome is a potentially life-threatening condition manifesting in the context of preeclampsia, which poses challenging diagnostic and management issues to the clinician.^1^ Its incidence is reported as 0.5-0.9% of all pregnancies and 10-20% of women with severe pre-eclampsia^2^ and causes significant mortality and morbidity, which increases with the severity of the syndrome.^3^

This study aims to determine the actual prevalence of HELLP syndrome and to evaluate the maternal-perinatal outcome associated with it so that early recognition and prompt management may improve maternal and fetal outcomes.

The primary objectives of this study are to find the prevalence of HELLP syndrome, Maternal outcome (acute kidney injury, abruptio placenta, postpartum haemorrhage, mortality), and Perinatal outcome (Intrauterine Fetal Demise/ Alive, APGAR at 5 minutes, gestational age at delivery). The secondary outcome was the mode of delivery, need of Intensive Care Unit, Neonatal admission rate.

## METHODS

We conducted a descriptive cross-sectional study at the Department of Obstetrics and Gynecology BP Koirala Institute of Health Sciences for one year from 1^st^ April 2017 to 30^th^ March 2018 after obtaining ethical clearance from the Institutional Review Committee of BPKIHS.

The sample size was calculated using the formula,

n = Z^2^ × (p × q)/e^2^

   = 1.96^2^ × (0.66 × 0.34) / (0.132)^2^

   = 0.862 / 0.017

   = 50.5

   = 51

where,

n = required sample size

p = According to a study done by Abroug et al,^4^ the prevalence of acute kidney injury in HELLP syndrome was 66%,

q = 1-p

e = margin of error, 13.2%

Z = 1.96 at 95% Confidence Interval

Adding a non-response rate of 10% the final sample size was 56. We enrolled 83 patients with HELLP syndrome during the study period.

The inclusion criteria were patient giving consent for participation and those who delivered in our hospital. Patient with HELLP syndrome who delivered outside and referred in the postpartum period was excluded because details of the neonate may not be available. Our hospital is a tertiary hospital so many patients with hypertension are referred from another centre. After admission, all hypertensive patients were investigated and managed according to the hospital protocol, and those who met the criteria for HELLP syndrome and meeting inclusion criteria were included in the study.

According to criteria developed at the University of Tennessee,^2^ this syndrome is characterized by the presence of all of the three following criteria: hemolysis (characteristic peripheral blood smear, serum lactate dehydrogenase > 600 U/l, total serum bilirubin > 1.2 mg/dl), elevated liver enzymes (serum aspartate aminotransferase > 70 U/l), and low platelet count (<100,000///l). Partial HELLP syndrome (PHS) is defined by the presence of one or two of the criteria. We had diagnosed HELLP syndrome according to these criteria. They were followed up till the discharge from the hospital and maternal and perinatal outcomes were recorded in the proforma.

Abruptio placenta was diagnosed by clinical assessment^5^ with complaints of vaginal bleeding, abdominal pain, hypotension, increased tone, or retroplacental clots during delivery of the placenta. Postpartum haemorrhage was defined as a blood loss of 500 ml or more within 24 hours after birth according to WHO.^6^ Acute kidney injury was diagnosed according to the AKIN^7^,^8^ criteria which are defined as Abrupt (within 48 h) reduction in kidney function currently defined as an absolute increase in serum creatinine of 0.3 mg/dL or more (>26.4 //mol/L) or a percentage increase in serum creatinine of 50% or more (1.5-fold from baseline) or a reduction in urine output (documented oliguria of < 0.5 mL/kg/h for >6 h). Statistical Package for Social Sciences version 21 was used for the analysis of the data and point estimate at 95% Confidence interval was calculated along with frequency and proportion for binary data and the analysis was done.

## RESULTS

The total number of deliveries within the study period was 11974 and there were 83 patients with HELLP syndrome. Hence, the prevalence of HELLP syndrome was 83 (0.69%) of total deliveries. Out of the 64 (77.1%) had partial and 19 (22.9%) had complete HELLP syndrome.

Table 1 shows the general variables of the study population (Table 1).

**Table 1:** Background characteristics of the study population (n=83).

Characteristics		n (%)
Age	Less than 19	16 (19)
	More than 20	67 (81)
Gravida	Primigravida	43 (51.8)
	Multigravida	40 (48.2)
Gestational age	Less than 34 week	50 (60.2)
	More than 34 week	33 (39.8)
Booked/Unbooked	Booked	17 (20.5)
	Unbooked	66 (79.5)
Gestation	Singleton	75 (90.36)
	Twin 8	(9.64)

Similarly 27 (32.5%) mothers required ICU stay, 56 (67.5%) did not. 22 (26.5%) required blood transfusion. One (1.2%) patient expired (Table 2).

**Table 2:** Maternal outcomes among HELLP syndrome patients.

Outcome		n (%)
Mode of delivery	Vaginal delivery	38 (45.8)
	Cesarean section	43 (51.8)
	Vacuum delivery	2 (2.4)
ICU* stay	Required	27 (32.5)
	Not required	56 (67.5)
Blood transfusion	Required	22 (26.5)
	Not required	61 (73.5)
At discharge	Improved	80 (96.4)
	Discharge on request	1 (1.2)
	Leaving against medical advice	1 (1.2)
	Expired	1 (1.2)

*ICU: Intensive Care Unit

Nine (47.37%) mothers had acute renal failure, 2 (10.53%) had abruptio placenta, 3 (15.79%) had pulmonary oedema and 4 (21.05%) had postpartum haemorrhage (Table 3).

**Table 3:** Maternal complications.

Complications	n (%)
Acute renal failure	9 (47.37)
Abruptio placenta	2 (10.53)
Pulmonary oedema	3 (15.79)
Postpartum haemorrhage	4 (21.05)
Retinopathy	2 (10.53)
Continuous seizure	1 (5.26)

*One patient had developed both abruption and postpartum haemorrhage and another one had both postpartum haemorrhage and acute renal failure

Eighty (87.9%) survival and 11 (12.09%) Intrauterine fetal death was seen (Table 4).

**Table 4:** Perinatal outcomes. (n=91)

Variables	n (%)
Alive	80 (87.9)
IUFD	11 (12.09)

Twenty-six (32.5%) newborn required neonatal admission. And there were 2 (2.5%) neonatal death. The most common cause of neonatal admission was the low birth weight (53.84%) followed by respiratory distress (26.92%). The mean birth weight was 2199.84gm± 703.46gm. Median APGAR scores at 1 and 5 minutes among live newborns were 7 and 8 respectively.

## DISCUSSION

HELLP syndrome is a life-threatening complication, considered to be a severe variant of hypertension in pregnancy, which is one of the major causes of a higher rate of maternal and perinatal morbidity and mortality. In this study prevalence of HELLP syndrome was 83 (0.69%) of total pregnancies which are comparable to the study done by Haram et al.^2^

All women enrolled were in the antenatal period in our study with an average gestational age at presentation being 36.17±3.55 weeks. And 60% of the patient had presented before 34 weeks of gestation. According to a study by Rahman & Wendon^9^ this disorder is diagnosed antenatally in 70% of the women. Mean maternal age in our study: 24.84±5.90 years which is comparable to the study conducted by Nigar Sadaf et al, i.e, 28.23±5.9 years.^10^ It has been shown that maternal mortality decreasing up to 1% with treatment although complications as placental abruption, acute kidney injury, subcapsular liver hematoma, retinal detachment occur in about 25% of women.

In this study maternal complications were seen among 22.9% of the patient and most common being an acute renal failure (47.37%) followed by postpartum haemorrhage (21.05%). This result is also similar to the study done by Nigar Sadaf et al.^10^ where acute renal failure was the most common maternal complication followed by abruptio placenta. Another study was done by Kestutis et al.^11^showed placental abruption in 14.7% of the cases with complete HELLP syndrome. Maternal mortality is as high as 37% as shown in the study done by P Tiwari et al. which is seen in only 1.2% of cases in our study.^12^ Less maternal mortality in our study could be due to the enrollment of fewer cases of complete HELLP syndrome (23%) because morbidity and mortality are more in complete than in partial HELLP syndrome.

In this study, 51.8% of the patient underwent cesarean section and the most common cause for cesarean was non-reactive fetal heart tracing (44%) followed by failed induction. Which is similar to other studies done by Nigar Sadaf et al^10^. Perinatal mortality and morbidity are considerably higher in this syndrome and are primarily dependent on the gestational age when the condition develops. The perinatal mortality rate related to HELLP syndrome is between 7.4% and 34%.^13^ Perinatal morbidity and mortality are associated with fetal growth restriction, oligohydramnios, non-reassuring fetal heart rate, preterm birth, low birth weight, birth asphyxia, stillbirth, and intrapartum death. And this is believed to be caused by placental insufficiency and generalized endothelial damage.^14^ In our study perinatal death is around 15% and the neonatal admission rate is 28.5% and a common cause for admission was low birth weight and respiratory distress, which is comparable to the study done by Zen et al. who reported perinatal mortality of 29% with HELLP syndrome.^15^ We have enrolled both partial and complete HELLP syndrome patients in this study which might have some influence on the maternal and perinatal outcome. If we would have taken the cases of complete HELLP syndrome result might have been more significant. This is an only descriptive study, it would have been better if we could have compared the findings between HELLP and non HELLP patients among hypertensive disorder.

## CONCLUSIONS

HELLP syndrome is a life-threatening complication of pregnancy, though its incidence is less it is associated with high maternal and perinatal morbidity and mortality. Hence early diagnosis and prompt management are required for the good maternal and perinatal outcome, as the delivery of the fetus is the best way to prevent complications.

## Conflict of Interest:

**None.**
